# Prevalence of Dyslipidemia in Undiagnosed Palestinian Men: A Cross-Sectional Study

**DOI:** 10.1155/2019/3473042

**Published:** 2019-10-13

**Authors:** Iyad Ali, Aya Kharma, Malak Samara, Samar Odeh, Nidal Jaradat, Abd Nasser Zaid, Mahdi Al Sayed Ahmad

**Affiliations:** ^1^Department of Biochemistry and Genetics, Faculty of Medicine and Health Sciences, An-Najah National University, Nablus, State of Palestine; ^2^Department of Pharmacy, Faculty of Medicine and Health Sciences, An-Najah National University, Nablus, State of Palestine

## Abstract

**Introduction:**

Dyslipidemia is the most important modifiable risk factor that leads to cardiovascular diseases. The screening for dyslipidemia in Palestine is not established in primary health care centers for healthy people. Our study aims to determine the prevalence of dyslipidemia among healthy undiagnosed adult men in Palestine in order to assess the need for screening and preventive programs for dyslipidemia.

**Materials and Methods:**

A cross-sectional observational study was carried out in 10 secondary schools at Nablus municipality (Palestine) from August 2017 to February 2018. The study included 140 teachers based on sample calculations. The age of participants ranged between 24 and 60 years. A questionnaire was used to collect demographic data about the lifestyle, past medical, and family histories. Serum lipid profile, and fasting blood glucose levels for each participant were measured. Lipoprotein levels were categorized based on the adult treatment panel III criteria.

**Results:**

The overall prevalence of dyslipidemia among Palestinian men was 66.4%. The most prevalent type of dyslipidemia was hypo HDL (*X* < 40 mg/dl, 59.3%), followed by hypertriglyceridemia (*X* ≥ 200 mg/dl, 20%). The prevalence of hyper LDL (*X* ≥ 160 mg/dl), hypercholesterolemia (*X* ≥ 240 mg/dl) was 8.5%, and 3.6%, respectively. About 15% of participants had glucose intolerance, and 4.3% had hyperglycemia (undiagnosed). Those with glucose intolerance, 13 (9.2%) have hypo HDL, while 9 (6.42%) have hypertriglyceridemia. On the other hand, out of hyperglycemic patients: 5 (3.5%) had hypo HDL, and 1 (0.7%) had hypertriglyceridemia.

**Conclusion:**

Around two-thirds of undiagnosed participants had at least one lipid abnormality. None of them were aware of having dyslipidemia. The prevalence of undiagnosed dyslipidemia was higher than the prevalence of undiagnosed glucose intolerance, and diabetes. This suggests that dyslipidemia plays a major role in developing diabetes. Hence, profound efforts should be done to manage and treat those with dyslipidemia, in order to prevent progression to type II diabetes mellitus.

## 1. Introduction

Cardiovascular disease is one of the leading notorious causes leading to increased mortality rates worldwide. Dyslipidemia is one of the most important modifiable risk factors for cardiovascular disease [1]. A high level of both total cholesterol (TC), and low-density lipoprotein cholesterol (LDL-C) are significant contributing factors for the progression of atherosclerosis—a disease due to plaque buildup in the endothelial walls of cells—which increases the risk for cardiovascular disease development [2].

Dyslipidemia is a disorder of lipid metabolism and is characterized by abnormal amounts of lipids circulating in the blood. This clinically includes elevated TC, TG, LDL-C, and decreased HDL-C levels. Dyslipidemia can be classified as primary (also called familial when present in more than one family member) and secondary. Primary dyslipidemia is caused by a specific genetic abnormality. Whereas, secondary dyslipidemia is primarily due to lifestyle, or secondary medical conditions like hyperthyroidism, or even cancer. In addition, dyslipidemia may be idiopathic (without a known cause) [1]. Acquired dyslipidemia is classified according to the type of elevated lipids.

The increasing prevalence of dyslipidemia has become a worldwide public health issue [2, 3]. The risk of mixed forms of dyslipidemia is also growing due to the increased prevalence of other metabolic diseases like diabetes mellitus, hypertension, and metabolic syndrome [3]. This potentially could occur due to dietary and lifestyle changes, reduced physical activity, and long-term sedentary work; all of which are regarded as major risk factors for dyslipidemia [2]. Hence, lifestyle modification remains the first step in the treatment of dyslipidemia. Nevertheless, it can be difficult to sustain, and achieve acceptable compliance in the elderly. In this case, treatment is best achieved in a combination of lifestyle changes, and drug therapy [4].

The prevalence of undiagnosed dyslipidemia in the adult male population is unknown. Many men are unaware of their lipid profile, leading to a high prevalence of patients with untreated lipid abnormalities. Our study aims to estimate the prevalence of dyslipidemia among undiagnosed Palestinian adult men, to assess the need for screening programs of dyslipidemia, in order to prevent progression into diabetes, and premature cardiovascular diseases.

## 2. Materials and Methods

This was a cross-sectional observational study that was carried out in 10 secondary schools at Nablus municipality (Palestine) from August 2017 to February 2018. The target population was Nablus high school teachers (225 teachers). The sample size was 140 teachers based on sample calculations (Rao soft calculator). The age of participants ranged between 24 and 60 years. Diabetic patients who take lipid-lowering medication, or who were previously diagnosed with dyslipidemia, were excluded from the study.

A structured questionnaire was used and divided into three parts. Part I contained items to assess the sociodemographic characteristics (age, marital status, income, and the number of family members). Part II contained items to assess behavioral risk factors such as smoking, physical activity patterns, and dietary behavior. Part III contained items to assess the past medical and family history.

Blood pressure, height, and weight were measured for all participants and body mass index (BMI) was calculated. All participants were aware of the benefits and harms of the study and agreed to sign a consent form. The total lipid profile (TC, HDL, LDL, and TG) and fasting blood glucose levels were measured. All participants were asked to fast for at least 12 h prior to the tests.

BMI was calculated by dividing the weight (in kilograms) by the square meters of height. BMI lower than 18.5 kg/m^2^ were classified as underweight, and between 18.5 and 24.9 kg/m^2^ as normal. BMI values between 25 and 29.9 kg/m^2^ were considered as overweight and value greater than 30 km/m^2^ were considered obese. Obese men were classified into three categories. Class I obese (BMI 30–34.9), class II obese (BMI 35–39.9), and class III obese (BMI ≥ 40) [5].

Blood pressure is considered normal when (*x* < 130/*x* < 85), pre-hypertensive when between 130–139 and 85–89, mildly hypertensive (HTN) 140–159 and 90–99, moderate HTN 160–179, and 100–109, and severe HTN >180/>110 [5].

Lipid disorders were defined according to National Cholesterol Education Program, Adult Training Program III (NCEP, ATP III) final report as TC ≥ 240 mg/dL, TG's ≥ 200 mg/dl, LDL-C ≥ 160 mg/dl, and HDL-C < 40 mg/dl. Glucose levels are considered normal when blood glucose <100 mg/dl, while values between 100 and 125 mg/dl are characterized as glucose intolerant, and hyperglycemic if blood glucose ≥126 mg/dl.

We visited 10 secondary schools. On the first visit, the purpose and methodology of the study were explained in detail to each participant eligible for the study. Each participant was given a consent form to sign before proceeding. Afterward, participants were interviewed using the study questionnaire. Their blood pressures, and fasting venous blood samples of 5 mL were collected by a lab technician and referred to Al-Najah National University Hospital laboratory for assigning sugar and lipid levels in the blood. Anthropometric measurements including height and weight of the participants were recorded with validated procedures. Data were analyzed using the Statistical Package for Social Sciences version 20. Descriptive analysis was performed, the relationship among nominal values was determined by the Pearson Chi-Square test, and Fisher exact test. All calculations were 2-tailed and a *P*-value < 0.05 was considered to be significant.

## 3. Results

A total of 140 adults participated in the study. All of them were males. The mean age of participants was 40.29 ± 9.27 years old (range: 24–60 years). Out of the 140 participants, 68 (48.6%) came from the rural region, and 56 (40%) subjects came from the urban region.

A total of 85.7% of the participants had never screened, nor done lipid profile tests before. Among the participants, 37.1% were smokers, and 27.9% had a family history of dyslipidemia. Based on the blood pressure measurements, 13.6% had high blood pressure (HTN) at the time of examination. Furthermore, 67 (47.9%) participants were overweight, and 49 (34.9%) participants were obese. Blood glucose levels were high (≥126 mg/dl) in 4.3% of them as Table 1 shows.

The overall prevalence of dyslipidemia was 66.4%. As shown in Table 2, the prevalence of hypercholesterolemia was 3.6%. Hyper LDL was present in 8.5% of participants. In contrast, the prevalence of hypo HDL was 59.3%, and the prevalence of hypertriglyceridemia was 20%. Two (1.4%) participants had very high LDL (≥190 mg/dl) and 1 participant (0.7%) had very high TG levels (≥500 mg/dl). The prevalence of borderline high TC, LDL, and TG were 27.1%, 30.7%, and 25%, respectively.

The prevalence of hyper LDL increased from 1.4% in the 24–30 age group to 2.14% in the 31–40 age group, and to 3.57% in the 41–50 age group. A decline to 1.4% was seen among the 51–60 age group. There was a significant influence of BMI on LDL levels (*P* = 0.038). In addition, a significant decrease in LDL levels was observed in smokers (*P* = 0.027). Furthermore, a significant difference between the prevalence of hypo HDL and the number of fast food meals per week (*P* = 0.002) was observed as seen in Table 3.

The overall prevalence of glucose intolerance and hyperglycemia was 15%, and, 4.3%, respectively. Among participants who were glucose intolerant, 13 (9.2%) had hypo HDL, and 9 (6.42v) had hypertriglyceridemia. Those with hyperglycemia, 5 (3.5%) of them had hypo HDL, and only 1 (.7%) participant had hypertriglyceridemia. Neither hyper LDL nor hypercholesterolemia was detected in glucose-intolerant participants or hyperglycemic participants.

Most of the glucose intolerance cases, 7 (5%) participants, were in the 31–40 age group. Meanwhile, participants who maintained exercise more than three times per week had the least prevalence of glucose intolerance 3 (2.14%). On the other hand, participants who did not exercise regularly had a higher, 10 (7.14%), prevalence of glucose intolerance. Hyperglycemia prevalence in smokers and nonsmokers was the same (3.14%). About (8.5%) of participants with a positive family history of diabetes had glucose intolerance (Table 4).

## 4. Discussion

The increasing prevalence of dyslipidemia has become a worldwide public health concern. The rates of dyslipidemia vary widely between ethnic, socioeconomic, and cultural characteristics of distinct population groups. This study was conducted in Palestine to determine the prevalence of dyslipidemia as well as associated risk factors. The overall prevalence of dyslipidemia was 66.4%. This nearly resembles results from a 2015 Iranian study which found that the overall prevalence of dyslipidemia was 51.8% [3]. Our findings were also in agreement with a study conducted on Korean adults, which reported the prevalence of dyslipidemia in the adult population was nearly 50% [3]. In contrast, dyslipidemia prevalence in Jordanian (75.7%), and Turkish adults (78.7% of men and 80.4% of women) has been reported to be higher than the prevalence observed among Palestinian, Iranian, and Korean participants [4, 6]. Additionally, a lower prevalence of dyslipidemia has been identified in Pakistan (32.7% of adults), [7] and Canada (14.0% of adults) [8]. The differences between our study and other studies may be due to different genetic predisposition, socioeconomic status, and lifestyle of the studied subjects.

Our study revealed that the overall prevalence of hypo HDL was 59.3% (the most prevalent type). A study conducted in the Northwestern Iranian urban on adults over 20 years old found that the prevalence of hypo-HDL was 63% in men [3]. Furthermore, Sawant et al. studied the prevalence of dyslipidemia in Indian adult men and found that hypo-HDL also was predominant (64.2%) [9]. The prevalence of hyper-LDL and hypertriglyceridemia in our study was 8.5% and 20%, respectively; which is again close to the prevalence of hyper LDL and hypertriglyceridemia among the Indian, [9] and Korean populations [10].

Regarding age distribution, the prevalence of dyslipidemia was in positive correlation with age and reached its maximum among the 41–50 age group. This indicates that there is a gradual deposition of cholesterol with age. A decline in dyslipidemia prevalence was seen among the 51–60 age group. This is probably because most of the participants from this age group were already diagnosed with diabetes and dyslipidemia; hence, excluded from the study. This comes in line with a study from the Brazilian city of Campos dos Goytacazes, which found a positive correlation between age and increasing lipid levels [11]. Cholesterol was more elevated in the age group of 40–49 years and decreased in older individuals. In the Nutrition and Health Survey in Taiwan, Chang et al. found that cholesterol levels were lower in men over 45 years of age than in other age groups [12]. In contrast to the Taiwanese study, a Turkish study showed that the prevalence of high TC, LDL, and TG increased with age, with the highest prevalence in the 46–65 years age group [6].

In the Turkish population, the prevalence of dyslipidemia components was evaluated based on location. Dyslipidemia rates were significantly lower in subjects living in villages than cities. In contrast, our study shows a higher prevalence of hyper LDL, hypo HDL, and hypertriglyceridemia in participants from villages than cities. However, hypercholesterolemia was more prevalent in participants living in cities. This is most likely due to differing lifestyles, and socioeconomic statuses.

We also observed an association between dyslipidemia, BMI, HTN, smoking, and fast food. There was a significant of relationship between BMI and LDL levels (*P* = 0.038). This was compatible with a study conducted in the United States which found a strong correlation between BMI and dyslipidemia [13]. On the other hand, we did not find a true correlation between HTN and blood lipid concentrations. Nevertheless, a study from Turkey associates the high prevalence of dyslipidemias with HTN. Another study conducted from Delhi, India, reported that both high LDL, and TC were found to be significantly greater among the hypertensive participants.

In our study, a statistically significant relationship between LDL and smoking was observed, but not between hypo HDL and smoking. In a Turkish study, the latter was different. The risk of developing low HDL-C was higher in smokers, but the risk of developing other types of lipid disorders did not differ in smokers, and nonsmokers [6]. Furthermore, a study from China noted: smokers were at a higher risk of developing dyslipidemia, and low HDL-C [2]. Our differing results may be explained by our smaller sample size compared with the Turkish and Chinese studies.

In our study, a significant difference between hypo HDL and the number of fast food meals per week was noticed. A study was done at the University of Hail, KSA, detected that LDL, and triglyceride levels were in positive correlation with the increased number of fast food meals consumed weekly. However, that study presented no relationship between HDL-C and fast food intake [14].

In addition, an inverse relationship was observed between regular exercise and dyslipidemia. Thus, appropriate community-based prevention strategies that emphasize the importance of physical activity, and behavioral changes are useful tools in controlling the dyslipidemia epidemic.

The prevalence of glucose intolerance and hyperglycemia among undiagnosed participants was 15%, and 4.3%, respectively. Soriguer et al. conducted a study about the prevalence of diabetes mellitus, and impaired glucose tolerance in an undiagnosed Spanish population. It was found that the prevalence of glucose intolerance and hyperglycemia in the Spanish population was 14.8% and 6.0%, respectively; which also came in line with our results [15].

The prevalence of diabetes is very high in Palestine (15.3%), which was not established by our study [16]. Our study found that the prevalence of undiagnosed diabetes was 4.3%, but the prevalence of dyslipidemia was much higher (66.4%). This explains the role of dyslipidemia in the development of type II diabetes mellitus. This means major efforts should be taken to manage and treat those with dyslipidemia in order to prevent the progression of diabetes mellitus.

Our study generally had advantages and disadvantages. Starting with the disadvantages, due to restricted time and budget, this study was conducted on a small sized population of 140 teachers, from 10 secondary schools in Nablus, Palestine. A larger population size would have given us more reliable results. Another disadvantage was the pool our participants were from, all participants were teachers who would more or less have similar lifestyles, and belong roughly to the same socioeconomic status. In the future, our study's scope could target a wider population. Nevertheless, our study also had advantages. For example, the dynamicity of the study took into consideration several contributory factors like age, exercise per week, income, and place of residence.

## 5. Conclusions

About 66% of Palestinian men 24 years of age and older were affected by at least one abnormal type of blood lipid. All of them were unaware of having dyslipidemia. This suggests that there is a defect in the primary health care system. Therefore, screening programs in Palestine need to be established in order to detect early signs of dyslipidemia. Early diagnosis allows early treatment to be conducted, which could potentially prevent dyslipidemia from developing cardiovascular, and endocrine diseases in the future.

## Figures and Tables

**Table 1:** Sociodemographic and anthropometric characteristics of participants.

Categories	Number (%)
*Age (years)*	
24–30	24 (17.6)
31–40	51 (36.5)
41–50	43 (30.7)
51–60	22 (15.6)
*Residency*	
Village	68 (48.6)
City	56 (40)
Refugee camp	16 (11.4)
*BMI*	
Underweight	1 (0.7)
Normal	23 (16.4)
Overweight	67 (47.9)
Class I obese	37 (26.4)
Class II obese	9 (6.4)
Class III obese	3 (2.1)
*Smoking?*	
Yes	52 (37.1)
No	88 (62.9)
*Family history of dyslipidemia?*	
Yes	39
No	101
*Blood pressure?*	
Normal	94 (67.2)
Prehypertension	27 (19.3)
Mild HTN	18 (12.9)
Moderate HTN	1 (0.7)
Severe HTN	0 (0)
*Previous lipid profile testing?*	
Yes	20 (14.3)
No	120 (85.7)
*Glucose level?*	
Normal	113 (80.7)
Glucose intolerance	21 (15)
Hyperglycemia	6 (4.3)

**Table 2:** Dyslipidemia prevalence in the participant based on laboratory results and the adult treatment panel (ATP III criteria).

Fasting lipoprotein levels based on ATP III		Level (mg/dL)	Frequency (*n*)	Valid (%)	Cumulative (%)
TC	Desirable	<200	97	69.3	69.3
	Borderline high	200–239	38	27.1	96.4
	High	≥240	5	3.6	100

LDL	Optimal	<100	34	24.3	24.3
	Above optimal	100–129	51	36.4	60.7
	Borderline high	130–159	43	30.7	91.4
	High	160–189	10	7.1	98.6
	Very high	≥190	2	1.4	100

HDL	Low	<40	83	59.3	59.3
	Normal	40–59	53	37.9	97.1
	High	≥60	4	2.9	100

TG	Normal	<150	77	55	55
	Borderline high	150–199	35	25	80
	High	200–490	27	19.3	99.3
	Very high	≥500	1	0.7	100

**Table 3:** Percentage of dyslipidemias according to demographic, socioeconomic variables, and family history.

Categories	High LDL	Hypercholesterolemia	Hypertriglyceridemia	Low-HDL
*Age (years)*				
24–30	1.42	0.71	2.85	11.42
31–40	2.14	0	7.14	21.42
41–50	3.57	2.85	7.85	17.85
51–60	1.42	0	2.14	8.57
*P*-value	*P* = 0.97	*P* = 0.95	*P* = 0.726	*P* = 0.294
*BMI*				
Underweight	0	0	0	0.71
Normal	0.71	0	1.42	7.85
Overweight	2.85	2.14	10	30.71
Obese				
Class I	3.57	1.42	7.85	17.14
Class II	.71	0	.71	2.14
Class III	.71	0	0	.71
*P*-value	*P* = 0.038	*P* = 0.144	*P* = 0.787	*P* = 0.402
*Smoking*				
Yes	2.14	0.71	7.85	25
No	6.42	2.85	12.14	34.28
*P*-value	*P* = 0.027	*P* = 0.367	*P* = 0.802	*P* = 0.141
*Family history of dyslipidemia*				
Yes	4.28	0.71	7.14	14.28
No	4.28	2.85	12.85	45
*P*-value	*P* = 0.301	*P* = 0.212	*P* = 0.885	*P* = 0.172
*Blood pressure*				
Normal	5	1.42	15	40.71
High normal	2.14	0.71	1.42	12.14
Mild HTN	1.42	1.42	2.85	5.71
Moderate HTN	0	0	0.71	0.71
Severe HTN	0	0	0	0
*P*-value	*P* = 0.054	*P* = 0.179	*P* = 0.536	*P* = 0.853
*Residency*				
City	3.57	14.28	12.14	28.57
Village	2.14	4.28	0.71	7.14
Refugee camps	2.85	12.14	7.14	23.57
*P*-value	*P* = 0.516	*P* = 0.815	*P* = 0.023	*P* = 0.939
*The number of fast food meal per week?*				
Never	1.42	0	5	15.71
1-2 times	7.14	3.57	12.14	29.28
3-4 times	0	0	2.85	14.28
*P*-value	*P* = 0.664	*P* = 0.738	*P* = 0.216	*P* = 0.002

**Table 4:** Associations between the glucose level and other parameters.

Demographic data and laboratory profile	Normal glucose level (%)	Glucose intolerance (%)	Hyperglycemia (%)	*P*-value
*Lipid profile*				
High-LDL	8.57	0	0	0.898
Low-HDL	46.42	9.28	3.57	0.318
High-TC	3.57	0	0	0.659
High-TG	13.57	5.71	0.71	0.14
*Age*				
24–30	15	2.14	0	0.05
31–40	30	5	1.42	
41–50	25.71	3.57	1.42	
51–60	10	4.28	1.42	
*Income*				
600–900 $USD	23.57	1.42	1.42	0.627
901–1200 $USD	40	7.85	1.42	
>1201 $USD	0.28	3.57	1.42	
*Exercise per week?*				
Never	30	7.14	2.14	0.634
1-2 times	33.57	5.71	0.71	
3-4 times	10	1.42	0.71	
7 times	7.14	0.71	0.71	
*A family history of diabetes*				
Yes	52.85	8.57	0.71	0.271
No	27.14	5.71	1.42	
*Smoking*
Yes	30	5	2.14	0.803
No	50.71	10	2.14	

## Data Availability

The data used to support the findings of this study are available from corresponding author upon request.

## References

[1] Farlex T. F. D. Hyperlipidemia. https://medical-dictionary.thefreedictionary.com/hyperlipidemia.

[2] Qi L., Ding X., Tang W., Li Q., Mao D., Wang Y. (2015). Prevalence and risk factors associated with dyslipidemia in Chongqing, China. *International Journal of Environmental Research and Public Health*.

[3] Mohammadbeigi A., Moshiri E., Mohammadsalehi N., Ansari H., Ahmadi A. (2015). Dyslipidemia prevalence in Iranian adult men: the impact of population-based screening on the detection of undiagnosed patients. *The World Journal of Men’s Health*.

[4] Bayram F., Kocer D., Gundogan K. (2014). Prevalence of dyslipidemia and associated risk factors in Turkish adults. *Journal of Clinical Lipidology*.

[5] Douglas G., Nicol F., Robertson C. (2013). *Macleod’s Clinical Examination E-Book*.

[6] Khader Y. S., Batieha A., El-Khateeb M., Al Omari M., Ajlouni K. (2010). Prevalence of dyslipidemia and its associated factors among Jordanian adults. *Journal of Clinical Lipidology*.

[7] Zahid N., Claussen B., Hussain A. (2008). High prevalence of obesity, dyslipidemia and metabolic syndrome in a rural area in Pakistan. *Diabetes & Metabolic Syndrome: Clinical Research & Reviews*.

[8] Petrella R. J., Merikle E. (2008). A retrospective analysis of the prevalence and treatment of hypertension and dyslipidemia in Southwestern Ontario, Canada. *Clinical Therapeutics*.

[9] Sawant A. M., Shetty D., Mankeshwar R., Ashavaid T. F. (2008). Prevalence of dyslipidemia in young adult Indian population. *The Journal Association of Physicians of India*.

[10] Koo B. K. (2012). Letter: prevalence of dyslipidemia among Korean adults: Korea national health and nutrition survey 1998-2005 (diabetes metab J 2012;36:43-55). *Diabetes & Metabolism Journal*.

[11] de Souza L. J., Souto Filho J. T. D., de Souza T. F. (2003). Prevalence of dyslipidemia and risk factors in Campos dos Goytacazes, in the Brazilian state of Rio de Janeiro. *Brazilian Archives of Cardiology*.

[12] Chang H. Y., Yeh W. T., Chang Y. H., Tsai K. S., Pan W. H. (2002). Prevalence of dyslipidemia and mean blood lipid values in Taiwan: results from the nutrition and health survey in Taiwan (NAHSIT, 1993-1996). *The Chinese Journal of Physiology*.

[13] Brown C. D., Higgins M., Donato K. A. (2000). Body mass index and the prevalence of hypertension and dyslipidemia. *Obesity Research*.

[14] Ibrahim Bashir A., Sma S., Ahmed M. Q. (2017). Study on the effects of fast food on the glucose and lipid profile aims to provide a platform to advocate a healthier lifestyle and better eating habits. *Journal of Pharmaceutical and Biological Science*.

[15] Soriguer F., Goday A., Bosch-Comas A. (2012). Prevalence of diabetes mellitus and impaired glucose regulation in Spain: the di@bet.es study. *Diabetologia*.

[16] World Diabetes Foundation Palestine National Diabetes Program. https://www.worlddiabetesfoundation.org/projects/west-bank-and-gaza-wdf15-1304.

